# IV Infiltration and Latex Allergy in an Infant: Early Recognition is Key to Prevent Lasting Disability

**DOI:** 10.21767/2472-0143.100028

**Published:** 2017-09-06

**Authors:** Brandon Lucke-Wold, Roopa Avula, Neal Shah, Gregory Borah, Carl Shrader

**Affiliations:** 1 Department of Neurosurgery, West Virginia University, School of Medicine, Morgantown, West Virginia, USA; 2 Department of Family Medicine, West Virginia University, School of Medicine, Morgantown, West Virginia, USA; 3 Department of Basic Pharmaceutical Science, West Virginia University, School of Pharmacy, Morgantown, West Virginia, USA; 4 Department of Plastic Surgery, West Virginia University, School of Medicine, Morgantown, West Virginia, USA

**Keywords:** Latex allergy, IV infiltration, Compartment syndrome, Early recognition, Favorable outcome

## Abstract

Latex allergy confounded by IV infiltration presents a serious problem for pediatric patients. If unrecognized, it can lead to serious neurologic deficits, loss of limb mobility, compartment syndrome, and ultimately lasting disability. Appropriate early recognition can prevent progression to these devastating outcomes. In this case report, we present an infant with IV infiltration and latex allergy. The case is used to highlight important clinical diagnostic criteria, treatment approaches, and how to prevent detrimental outcomes. We provide a detailed review of the literature and highlight the key teaching points in a reader-friendly reference table.

## Introduction

Acute injury to the upper extremity secondary to IV infiltration in the pediatric population is a rare but important complication that must be recognized by the healthcare community in order for appropriate clinical management. This complication can potentially lead to catastrophic sequelae including permanent loss of range of motion or use of the arm, neurologic deficits, myonecrosis, decrease in ability to perform activities of daily living, and loss of limb to amputation [1]. Furthermore, the infiltration can lead to permanent scarring, which is potentially devastating for young children. Though there is a good deal of literature discussing acute compartment syndrome (ACS) in children due to fractures of the upper and lower extremities, cases of ACS in the upper extremity due to iatrogenic causes are greatly underrepresented in the current literature [2]. Infiltration can be exacerbated by hypersensitivity reactions such as latex allergies since the immune system is already primed, thereby increasing edema. This is especially worrisome since adhesive tape and rubber catheters are commonly used in the hospital.

We present herein a case of a serious adverse reaction due to IV infiltration in a 10-month old female infant worsened by an underlying latex allergy. We discuss the presenting symptoms in order to highlight early recognition of this potentially devastating reaction. Other key learning points discussed include how to manage this complication in the pediatric population, preventative approaches including careful monitoring of IV site with documentation and appropriate screening for potential latex allergies, and a detailed review of the current literature for iatrogenic ACS. This report is intended to increase awareness about an important complication that can be effectively managed if recognized and addressed in a timely manner.

## Case Description

**Figure 1:**
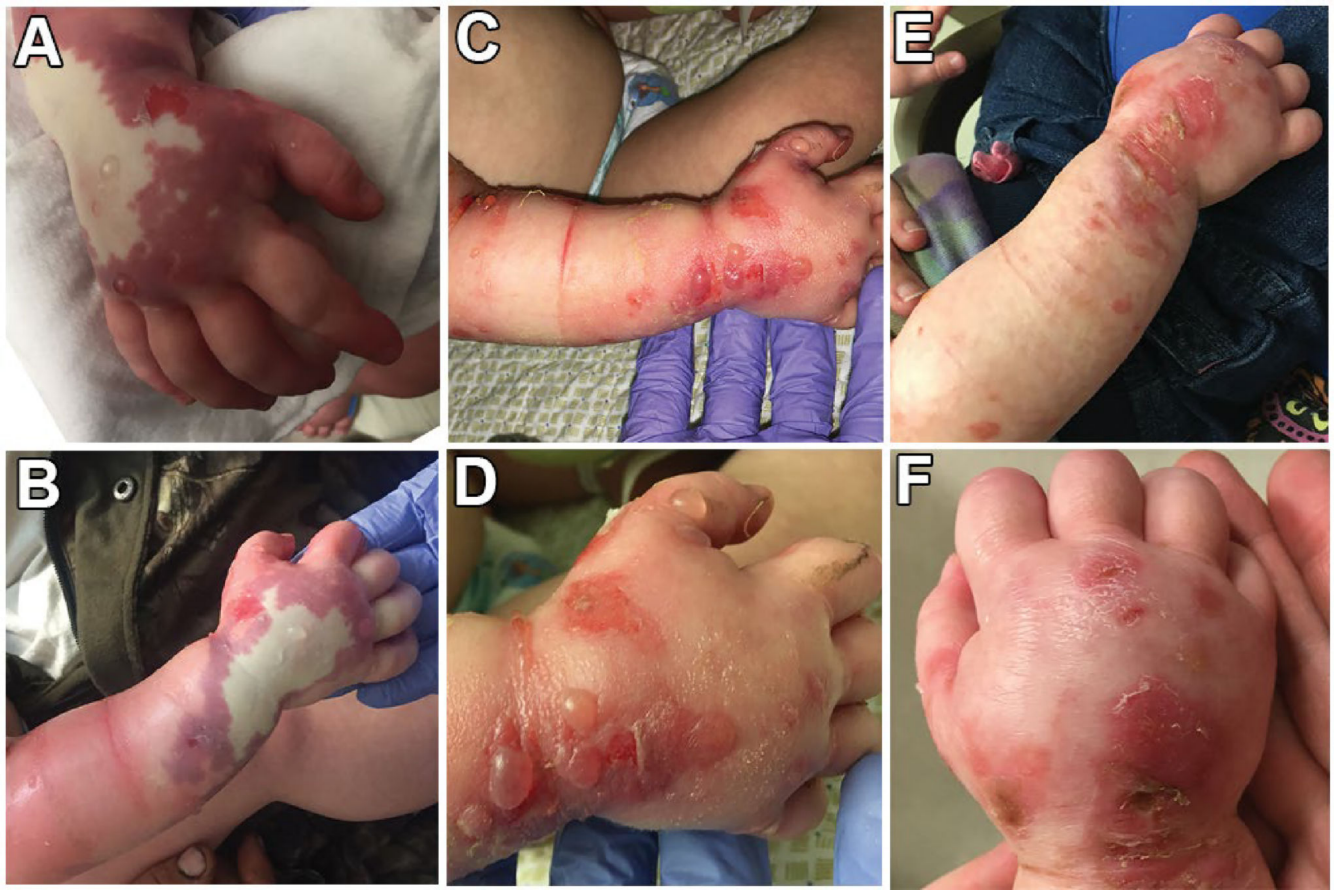
Day 1: after removal of the IV line, the dorsum of the right hand became pale and necrotic (A) and bullae, tender erythema, and edematous bruising was present and spreading on the right mid-humerus and axilla (B), potentially suggesting compromised blood supply. Diphenhydramine, acetaminophen, ibuprofen and lidocaine therapy was initiated. Pulse and compartment pressures were stable, prompting conservative management. Day 2: The dorsal non-blanching area resolved and erythema did not spread past the antecubital region (C) but bullae persisted alongside sloughing of skin (D). The patient was discharged on Day 3 with placements of occlusive dressings and a scheduled follow-up visit. Day 7: Bullae and erythema was markedly reduced on both the mid-humerus (E) and dorsum of hand (F), and the infant showed improved movement of the right arm.

This is a case of a 10-month-old female infant who presented to the emergency department (ED) for vomiting and decreased per os (p.o.) intake secondary to rhinovirus. After attempts to feed the infant p.o. failed in the ED, she was admitted overnight. Vital signs and labs were normal, prompting treatment with supportive care. The infant received a 20 ml/kg bolus of normal saline in the ED via peripheral IV in the dorsum of her right hand, and once admitted was started on 1.5× maintenance IVF consisting of D5W 1/2 Normal Saline and 20 KCl at 55 ml/hr. Overnight, she tolerated the IV fluids well and was able to take a bottle. Urine output measured 62 ml/kg. IV fluids were to be discontinued that morning. At morning rounds, the mother reported that the patient did well overnight, but mentioned her shirt became wet after holding the infant. The IV line was examined and no leaks or abnormalities were noted. The IV was flushed and removed by the nurse at 0730 without incident. 30 minutes after removal of the IV, the patient began to have an acute reaction at the IV insertion site extending up the right arm. The patient's right hand and forearm appeared erythematous and tense, with eruption of bullae observed on dorsum and palm of the hand. The bullae and erythema extended up the forearm and upper extremity to the chest wall. The arm also had diffuse edema from mid-humerus to hand. Furthermore, a large white lesion in the center of the dorsum of the right hand with surrounding deep purple bruising was noted concerning for necrosis (Figure 1). Bullae were cultured, and the infant was immediately started on diphenhydramine, pediatric acetaminophen, and ibuprofen p.o. 4% topical lidocaine cream was applied directly to the arm. Both the orthopedic as well as plastic surgery teams were consulted at this time.

The patient had no recorded allergies, but the mother of the infant had a severe latex allergy and siblings in the family had documented sulfa allergies. Based on family history, infant was placed on latex allergy alert. At 1-hour post IV removal, physical exam showed a tender swollen right arm. Radial pulse was palpable, and capillary refill was under 3 seconds. Additionally, the white central lesion did not blanche, but was compressible. After an additional 30 minutes, the erythema and induration had regressed from the chest wall and was only seen from mid-humerus to the hand. Compartment pressures were stable. After consulting with plastics and orthopedics the decision was made not to perform fasciotomy but to continue with conservative management. In addition to the aforementioned medical therapy, the patient's mother was instructed to keep the patient's arm elevated. The patient's arm was cleaned, treated with Bacitracin ointment, and covered with Xeroform and a loose Kerlix overlay. The care team followed the patient with serial visits throughout the remainder of the day and night, noting improvement throughout. By the following morning, the non-blanching white area had resolved, bullae were still visible, and the patient appeared to be improving (Figure 1). The patient was able to be discharged to home on day 3 of admission, and followed at the outpatient clinic by the primary team as well as plastic surgery.

Differential diagnoses for this encounter included atypical hypersensitivity reaction to latex, acute reaction to IV infiltration complicated by acute compartment syndrome (ACS), and compartment syndrome due to infection or necrotizing fasciitis. Gram cultures grew no organisms, including up to 3 days later. 5 days after discharge, mother reported significant improvement, and that the infant was tolerating dressing changes well. She was also moving her right arm more. Edema was still noted, but bullae and erythema were improving and epithelization was progressing well. The plastics team recommended Vaseline with Xeroform changes (Figure 1). 11 days after discharge, the parents reported continued improvement and increasing ROM in patient's right arm (Figure 1). Follow-up at 5 weeks showed continual improvement, but not complete return to baseline (Figure 1). Referral was made for physical therapy for continued help with range of motion and use of right hand and arm.

## Discussion

This case illustrates the potential severity of acute reaction to IV infiltration in the upper extremity in a pediatric patient. Fortunately, this patient did well with conservative management. Early recognition of symptoms with immediate treatment, urgent consults, serial team follow-up visits, and consideration of possible hypersensitivity to latex all played important roles. Recognition of the seriousness of IV infiltration, including the possibility for acute compartment syndrome, can be life-saving in this population.

Cases of pediatric patients with similar presentations are scant in the literature. Known complications of IV infiltration include infection, compartment syndrome, necrotizing fasciitis, myonecrosis, and tissue damage [3]. Acute compartment syndrome of the upper extremity most often results from fractures and major trauma, including crush injuries and burns [1]. However, iatrogenic causes have also been reported and include IV infiltration, adverse parenteral drug reactions, and other hypersensitivity reactions. The clinically accepted and validated early signs of compartment syndrome include: pain out of proportion to injury, edema, and diminished pulses [3]. Inability of infants to communicate pain clearly is an obvious pitfall to using this approach. Additionally, infants with atopic reactions may not yet have had previous exposures or known reactions, so careful history must be obtained from the family. Knowledge about these complications and successful training of the healthcare team to recognize them is necessary prior to using IV on the upper extremity in children.

Latex can be found in the tips of rubber IV catheters, rubber stopcocks, and adhesive tape [4]. Latex allergy also is associated with hypersensitivity to certain fruits and foods including avocado, kiwi, chestnuts, and banana [5]. This requires nurses, physicians, and the dietary team to be trained on how to recognize allergies in children who are not able to successfully communicate about pain or discomfort. Part of this training is to know how to take a careful history from the family asking about food allergies and family history of atopic reactions prior to initiation of IV administration. Parents may not think to mention these allergies and therefore must be specifically asked.

IV infiltration is defined by extravasation of material outside of the intended vein, causing potentially harmful sequelae. Mechanisms of infiltration may include things such as the patient dislodging the catheter (especially in pediatric populations), spontaneous disruption of the catheter, or osmolarity or chemical content of the infusion substance. The end result is increased interstitial pressure with loss of perfusion, which can lead to compartment syndrome, rapid deterioration of tissue, and myonecrosis [3]. While it has been said that other warning signs may include slowing of infusion rate or decreased blood return during aspiration, Krenn, et al. advise against relying on these signs alone, pointing out that they may only occur once pressures have elevated significantly or may not occur at all [6]. For example, in the above case, the initial clue was the mother reporting that her shirt was wet. The infant was not crying or showing signs of pain initially, and there were no other obvious signs of infiltration while the IV was still inserted.

While some studies have been conducted regarding IV infiltration injuries in the upper extremity of pediatric patients, current literature is scant at best. Most published studies describe infiltration associated with long bone fracture. Prasarn et al. conducted a retrospective review of pediatric cases of ACS in the upper extremity in the absence of a long bone fracture, demonstrating that the majority of cases (58%) had resulted from iatrogenic causes [2]. About 30% of these cases were due specifically to IV infiltration in the hand or forearm alone, and 50% of these resulted in abnormal functional outcome even amputation in some cases. Delay in treatment is an important contributing factor to poor outcome, particularly if diagnosis is missed or delayed. Grottkau et al. point out that pediatric cases of ACS with atraumatic, nonfracture-related etiologies tend to have the longest delays in surgical decompression [7]. This further highlights the importance of early symptom recognition in order to decrease time to treatment and decrease potentially serious outcomes.

Recognizing symptoms of ACS in young children and infants can be particularly challenging. Accepted symptoms in adults include: pain, paresthesia, pain with passive stretch, loss of 2-point discrimination, pallor, and decreased pulses, but these are unreliable in young children or those who cannot communicate effectively [7]. Rather, it has been suggested that more reliable indicators may include signs such as: greater need for analgesia (i.e., increased frequency or dose requirements), crying infant that cannot be comforted, and obvious signs of pain such as grimacing. Grottkau et al. point out that due to the subjective nature of these signs, high clinical suspicion should warrant immediate measurements of compartment pressures to avoid delays in treatment [7].

The patient we present in our case had a very good outcome thanks to recognition of symptoms, consultation with appropriate care management teams, and in this case conservative management with very close follow-up. As the initial wounds began to heal, the patient was followed in outpatient clinic by primary care, plastic surgery, and physical and occupational therapy, and has progressed very well. Key learning points are highlighted in Table 1. This case demonstrates that not all cases of suspected ACS require surgical decompression or have catastrophic outcomes, but the potential of missed or late diagnosis and treatment can result in loss of function or loss of limb. As such, we concur with Grottkau et al. that a national survey of diagnosis, treatment, and outcomes of upper extremity ACS cases in the pediatric population should be undertaken.

## Conclusion

Typical symptoms of IV infiltration (site erythema, bullae formation, non-blanchable areas) are shared by latex or drug hypersensitivity and necrotizing fasciitis, and may present more subjectively in pediatric patients (inconsolable crying, increased pain relief requirements, reduced extremity motion). Early recognition of this easily treatable reaction is crucial to prevent life-threatening sequelae, which includes acute compartment syndrome and potential amputation. An accurate food and medication allergy history, knowledge of vesicant drugs, avoidance of peripheral line usage, prompt rule-out of infectious etiology, and early infusion discontinuation must be done. Monitoring of the infiltrated site and proper wound care reduces the risk of IV infiltration occurrence. With prompt diagnosis, conservative management with antihistamines, pain relief, extremity elevation, and dressing changes can lead to favorable outcomes.

## Figures and Tables

**Table 1:** Recommendations to decrease incidence of extravasation injuries in pediatric patients.

S. No.	Recommendations to decrease incidence of extravasation injuries in pediatric patients
1	Design evidence-based protocol for hospital staff with clear roles of nurses and physicians for: proper surveillance, monitoring, staging and reporting of suspected IV infiltration in pediatric patients
2	Careful history taking, including food and fruit allergies in all pediatric patients to uncover possible latex sensitivities or other nonobvious hypersensitivities that may exacerbate an infiltration injury (for example, from the adhesive on the tape used to secure the catheter)
3	Use of a large vein in the distal forearm for peripheral IV access, since the majority of infiltration injuries are located on the dorsum of the hand and antecubital fossa
4	Clear guidelines regarding specific drugs or vesicants that should only be given via central access versus a peripheral IV line
5	Careful hourly visual monitoring and inspection of IV sites by nurses, including uncovering dressings and comparing with contralateral extremity, with specific and clear documentation, including but not limited to: time, site, specific wording to describe presence or absence of fluid leakage, swelling, or other signs of extravasation
6	Immediate discontinuation of IV at earliest signs of infiltration with proper technique, and immediate notification to and inspection by physician
7	Frequent IV site changes (at least every 48–72 hours) in order to decrease risk of IV infiltration or infection
8	Education to nurses and physicians of: staging of IV infiltrations, differences in vesicants and nonvesicants, antidotes to common vesicants, when to aspirate rather than flush the line for removal (for example, avoid flushing the line if allergy or reaction to vesicant is suspected)
9	Adverse outcomes reporting to keep track of iatrogenic errors, which will ideally lead to reduction in such errors over time
10	Conservative management when compartment pressures are low/stable and symptoms are discovered early, with serial follow-up hourly
